# Special Issue: “Genomics and Models of Nerve Sheath Tumors”

**DOI:** 10.3390/genes11091024

**Published:** 2020-09-01

**Authors:** Angela C. Hirbe, Rebecca D. Dodd, Christine A. Pratilas

**Affiliations:** 1 Siteman Cancer Center, Washington University School of Medicine, 660 South Euclid Avenue, Campus Box 8076, St. Louis, MO 63110, USA; 2 Holden Comprehensive Cancer Center, Carver College of Medicine, University of Iowa, 285 Newton Road, Iowa City, IA 52242, USA; 3 The Sidney Kimmel Comprehensive Cancer Center, Johns Hopkins University School of Medicine, Baltimore, MD 21287, USA

**Keywords:** genomics, mouse models, NF1, nerve sheath tumors

Nerve sheath tumors arising in the context of neurofibromatosis type 1 (NF1) include benign tumors such as cutaneous, diffuse and plexiform neurofibromas; atypical neurofibromas or atypical neurofibromatosis neoplasms of uncertain biological potential (ANNUBP); and the aggressive soft tissue sarcoma, the malignant peripheral nerve sheath tumor (MPNST). Even benign tumors often represent a significant cause of morbidity for many patients, due to disfigurement, disability, or organ dysfunction. MPNST are aggressive, often metastasize, and are often lethal. An expanding body of literature related to genomic alterations common to MPNST, signaling events that regulate tumorigenesis, and novel models that recapitulate the human tumor, has informed novel therapeutic approaches. Despite numerous clinical trials, curative responses to treatment remain limited for patients with this malignancy. Here, we have compiled a series of articles that focus on the genomics of MPNST and the latest models generated to study these tumors.

Included in this Special Edition are six manuscripts that present original research highlighting novel therapeutic strategies, models, and genomic findings, as well as a whitepaper describing consortium efforts to genomically characterize MPNST. Staedke et al. [1] present a chemoprevention strategy repurposing two drugs already in clinical use for other indications (mebendazole and cyclooxygenase-2 inhibitors), utilizing one of the most commonly used preclinical models for preclinical testing of MPNST, the *cis Nf1+/−;Tp53+/−* (*NPcis*) mouse model [2,3]. In these studies, they report that mebendazole reduces levels of RAS-GTP, delays the formation of solid malignancy in at-risk mice, and increases survival. Further clinical studies are needed to validate the potential of this strategy in humans, but the study demonstrates the feasibility of a prevention strategy for NF1-associated malignancy. The article by Scherer et al. highlights newer mouse models of MPNST that use somatic CRISPR/Cas9 tumorigenesis to generate genomically-matched tumors in different background strains of wild-type mice [4]. This is the first study to systematically evaluate the impact of host strain on CRISPR/Cas9-generated mouse models and identifies several key strain-dependent phenotypes, including impacts on tumor onset and the tumor immune landscape. Moon and Tompkins et al. performed a comprehensive genomic analysis of multiple areas from within a single large MPNST. These authors identify varied genomic profiles within each area, highlighting the need for further studies on intra-tumoral heterogeneity in order to truly understand the genomic composition of any given tumor [5]. Such studies are critical to aid in our understanding of tumor and patient responsiveness and non-responsiveness to a range of therapies. Miller et al., on behalf of the Genomics of MPNST (GeM) consortium, present a whitepaper describing the composition, design, and analysis plan of this consortium, founded by the NF Research Initiative at Boston Children’s Hospital. These authors have aimed to perform the most comprehensive genomic analysis of the largest cohort of MPNST to date, data from which will be shared on an outward-facing web-based interface made available to other investigators, in order to accelerate collaborative and therapy-directed research [6]. Grit et al. describe their experiments using reverse phase phospho-proteome array (RPPA) analysis of murine MPNST models to determine mechanisms of resistance to commonly-used therapies, including DNA damaging agents (doxorubicin) and kinase inhibitors (MET and MEK inhibitors). These authors observed profound signaling plasticity in treated tumors, with key activation of the AXL and NFkB pathways that were associated with the development of resistance [7]. Banerjee et al. set out to design an integrative approach that combined multiple transcriptomic and genomic datasets, the analysis of which would be poised to identify new therapeutic avenues in MPNST. Gene expression data from four independent studies were integrated and analyzed using a transfer learning-inspired approach to identify latent variables (LV)—groups of genes derived from larger repositories of gene expression datasets that exhibit common transcriptomic patterns relevant to a specific subset of samples—and thereby uncover previously unknown biology. To assess the biological underpinnings of uncharacterized LVs, a tumor immune cell deconvolution analysis was used, which indicated the presence of activated mast cells and M2 macrophages in all tumor types, as well as CD4 memory T-cells [8]. The findings uncovered using these computational approaches suggest potential biological signatures rich for experimental and clinical investigation.

The Special Edition also includes three review articles. Lemberg et al. have compiled a collated summary of sequencing efforts in MPNST published in the past two decades, using a total of 12 studies to summarize the range of incidences of the most common mutations in *NF1, CDKN2A, TP53, EED* and *SUZ12*. In this article, the authors review the initial findings of *NF1* as the gene responsible for neurofibromatosis type 1, its function as a RAS-GTPase-activating protein (RAS-GAP), and the spectrum of alterations in *NF1* found in human disease. They then further summarize 16 additional genomic studies, covering 10 other recurrently altered genes, including *BRAF, MET, EGFR, TYK2, ATRX* and others [9]. Williams and Largaespada review the range of published MPNST model systems, including genetically-engineered mouse models (GEMM), the genes involved, and the limitations of these models. They elaborate on the commonly used *NPCis* mouse, its genetic design, and the tumors that develop in these mice, as well as human-derived cell lines and xenografts. The use of synthetic lethality screens to identify combination drug therapies is explored, as are dysregulated signaling pathways that represent targets for molecularly based therapies [10]. The review article by Zhang et al. discusses the current biological understanding of polycomb repressive complex 2 (PRC2) loss in MPNST, which is a frequently-mutated pathway in these tumors. This article also highlights PRC2 function in normal Schwann cell development and nerve injury repair, in addition to discussing potential therapies that target PCR2 deficiency in tumor cells [11].

In conclusion, the articles that we have assembled in this Special Edition on Genomics and Models of Nerve Sheath Tumors highlight the most recent scientific advances on the genomic composition of malignant peripheral nerve sheath tumors and review novel efforts to model and study these tumors. While a wide range of benign, borderline and malignant nerve sheath tumors affect individuals with neurofibromatosis type 1, our collection of articles here focuses primarily on malignant nerve sheath tumors and underscores the pressing need for novel therapies. As genomic and transcriptomic capabilities continue to advance at an impressive pace, the hope is that an improved understanding of the genetics, and therefore the pathobiology, of these tumors, will ultimately lead to effective therapies that result in deeper and more durable responses, and therefore improved survival rates for these patients.
